# Quinine in Cholera Infantum

**Published:** 1882-09

**Authors:** Otis F. Manson


### Quinine in Cholera Infantum.

Called to see a patient *at night*, along with the means already advised, you will now make an important addition to your treatment. We usually, unless the case is very urgent, postpone the administration of the grand remedy, quinine, until the late hours of night. Then the violent excitement and commotion of the exacerbations have passed. The stomach is then not so irritable. Quinine, a *sedative and narcotic*, is assisted in its action by the physiological tendency of the nervous system to repose in the night season, and you have ample time to exhibit enough of it in order to prevent the threatened exacerbations of the next day. I am sure that the remedy is better borne, and produces its salutary effects in the most perfect manner, when exhibited in the late hours of night. At midnight, then, we can commence its use. To an infant of six months of age and under, we give a grain of sulphate of quinine with a few grains of white sugar, diffused in a teaspoonful of cold water. To a child of twelve months, we give two grains of quinine, and to one of eighteen months, three grains. If the dose is immediately rejected, we repeat it over and over again every half hour. After a few repetitions enough will be absorbed by the mucous membrane of the mouth and stomach, or by the former alone, if it is not even swallowed, to bring the little patient fully under its influence. If the first dose is, however, entirely retained, we allow the patient to rest for three or four hours. We then repeat the dose, and continue to repeat it until the thermometer in the axilla and the finger on the pulse indicate that rapid sedation is ensuing. In the large majority of cases these effects will follow from the administration of the sulphate. The pulse will become slower and less active and bounding; the head will become cooler; and the extremities, if previously below the normal temperature, will become warmer. Not only this, but the vomiting will become less frequent, or will often entirely cease. After the first dose of quinine has been absorbed, the bowels will become more quiet, and the renal secretion more copious. The little sufferer will become tranquil, and fall asleep, sometimes for hours without awaking; but it can be easily aroused if necessary. The narcotism produced by quinine, in this respect, is unlike the stupor produced by opium; and, besides this, instead of having a tendency to produce congestion of the brain, like opium, it has, beyond all other remedies, the power of removing an excess of blood from the cerebral vessels. In five or six hours the administration of quinine has begun, in the large proportion of cases, seen early in their course, the fever will have disappeared. When this occurs, cease medication for the day. On the next afternoon or night a slighter exacerbation will often make its appearance, and this may recur for two or three nighes thereafter. In this same case, repeat the quinine in similar or diminished doses, giving it more freely in direct proportion to the violence of the fever. At the same time we continue the calomel, or employ blue mass, until the presence of pure, healthy bile in the dejections is perfectly evident. Now that the fever has vanished, you may associate opium in minute doses with the mercurials. If blue mass is given, have it triturated in a teaspoonful of simple syrup, and add the laudanum to it.—*Otis F. Manson, M. D., in Trans. Virginia Med. Society and Southern Practitioner.*

